# Comprehensive transcriptional atlas of human adenomyosis deciphered by the integration of single-cell RNA-sequencing and spatial transcriptomics

**DOI:** 10.1093/procel/pwae012

**Published:** 2024-03-15

**Authors:** Tao Chen, Yiliang Xu, Xiaocui Xu, Jianzhang Wang, Zhiruo Qiu, Yayuan Yu, Xiaohong Jiang, Wanqi Shao, Dandan Bai, Mingzhu Wang, Shuyan Mei, Tao Cheng, Li Wu, Shaorong Gao, Xuan Che

**Affiliations:** Department of Obstetrics and Gynecology, Affiliated Women and Children Hospital of Jiaxing University, Jiaxing 314000, China; Shanghai Key Laboratory of Maternal Fetal Medicine, Clinical and Translational Research Center of Shanghai First Maternity and Infant Hospital, Frontier Science Center for Stem Cell Research, School of Life Sciences and Technology, Tongji University, Shanghai 200092, China; Key Laboratory of Animal Bioengineering and Disease Prevention of Shandong Province, College of Animal Science and Technology, Shandong Agricultural University, Taian 271018, China; Shanghai Key Laboratory of Maternal Fetal Medicine, Clinical and Translational Research Center of Shanghai First Maternity and Infant Hospital, Frontier Science Center for Stem Cell Research, School of Life Sciences and Technology, Tongji University, Shanghai 200092, China; Women’s Hospital, School of Medicine, Zhejiang University, Hangzhou 310013, China; Postgraduate training base Alliance of Wenzhou Medical University, Wenzhou Medical University, Wenzhou 325035, China; Department of Obstetrics and Gynecology, Affiliated Women and Children Hospital of Jiaxing University, Jiaxing 314000, China; Department of Obstetrics and Gynecology, Affiliated Women and Children Hospital of Jiaxing University, Jiaxing 314000, China; Jiaxing University Master Degree Cultivation Base, Zhejiang Chinese Medical University, Hangzhou 310053, China; Shanghai Key Laboratory of Maternal Fetal Medicine, Clinical and Translational Research Center of Shanghai First Maternity and Infant Hospital, Frontier Science Center for Stem Cell Research, School of Life Sciences and Technology, Tongji University, Shanghai 200092, China; Shanghai Key Laboratory of Maternal Fetal Medicine, Clinical and Translational Research Center of Shanghai First Maternity and Infant Hospital, Frontier Science Center for Stem Cell Research, School of Life Sciences and Technology, Tongji University, Shanghai 200092, China; Postgraduate training base Alliance of Wenzhou Medical University, Wenzhou Medical University, Wenzhou 325035, China; Postgraduate training base Alliance of Wenzhou Medical University, Wenzhou Medical University, Wenzhou 325035, China; Shanghai Key Laboratory of Maternal Fetal Medicine, Shanghai Institute of Maternal-Fetal Medicine and Gynecologic Oncology, Clinical and Translational Research Center, Shanghai First Maternity and Infant Hospital, School of Life Sciences and Technology, Tongji University, Shanghai 200092, China; Shanghai Key Laboratory of Maternal Fetal Medicine, Clinical and Translational Research Center of Shanghai First Maternity and Infant Hospital, Frontier Science Center for Stem Cell Research, School of Life Sciences and Technology, Tongji University, Shanghai 200092, China; Department of Obstetrics and Gynecology, Affiliated Women and Children Hospital of Jiaxing University, Jiaxing 314000, China; Postgraduate training base Alliance of Wenzhou Medical University, Wenzhou Medical University, Wenzhou 325035, China

**Keywords:** adenomyosis, single-cell RNA sequencing, spatial transcriptomics, endometrial-myometrial junction, progenitor cells

## Abstract

Adenomyosis is a poorly understood gynecological disorder lacking effective treatments. Controversy persists regarding “invagination” and “metaplasia” theories. The endometrial-myometrial junction (EMJ) connects the endometrium and myometrium and is important for diagnosing and classifying adenomyosis, but its in-depth study is just beginning. Using single-cell RNA sequencing and spatial profiling, we mapped transcriptional alterations across eutopic endometrium, lesions, and EMJ. Within lesions, we identified unique epithelial (*LGR5*^+^) and invasive stromal (*PKIB*^+^) subpopulations, along with *WFDC1*^+^ progenitor cells, supporting a complex interplay between “invagination” and “metaplasia” theories of pathogenesis. Further, we observed endothelial cell heterogeneity and abnormal angiogenic signaling involving vascular endothelial growth factor and angiopoietin pathways. Cell-cell communication differed markedly between ectopic and eutopic endometrium, with aberrant signaling in lesions involving pleiotrophin, TWEAK, and WNT cascades. This study reveals unique stem cell-like and invasive cell subpopulations within adenomyosis lesions identified, dysfunctional signaling, and EMJ abnormalities critical to developing precise diagnostic and therapeutic strategies.

## Introduction

Adenomyosis is a complex and enigmatic gynecological disease characterized by the presence of endometrial tissue within the myometrium (Benagiano and Brosens, 2006). This condition presents many clinical challenges, including severe pelvic pain, abnormal uterine bleeding, and infertility, and poses a substantial burden on the quality of life for affected individuals (Chapron et al., 2020; Martire et al., 2020). The estimated prevalence of adenomyosis ranges from 5% to 70%, with approximately 20% of individuals of reproductive age with a uterus being diagnosed with this condition (Kho et al., 2021; Yu et al., 2020). Despite its prevalence and clinical significance, our understanding of the molecular underpinnings of adenomyosis remains incomplete and thereby therapeutic options are limited. Hysterectomy, though definitive, precludes fertility (Dason et al., 2021). While hormonal therapies, inducing temporary amenorrhea, offer only transient relief (Kobayashi, 2023; Moawad et al., 2023). Consequently, it is crucial to thoroughly understand the molecular underpinnings of adenomyosis to guide more efficacious therapeutic strategies.

Two primary hypotheses have been put forth to interpret the comprehensive molecular mechanisms underlying adenomyosis, but are controversial (Stratopoulou et al., 2021). The “invagination” theory has been proposed to result from altered endometrial cells invading the myometrium, crossing an injured or abnormal junctional zone, and subsequently establishing ectopic adenomyotic lesions. Alternative hypotheses proposed that the “metaplasia” theory advocating for the conversion of Mullerian remnants or adult stem cells into adenomyotic tissue (Donnez et al., 2018; García-Solares et al., 2018). The invagination theory implicates abnormal inward growth of endometrium, the metaplasia theory conversely suggests adenomyosis originates from stem cell dysregulation within the myometrial compartment (Guo, 2020). Furthermore, adenomyosis presents a complex process involving a series of molecular changes associated with inflammation, invasion, angiogenesis, and abnormal immune microenvironment (Vannuccini et al., 2017). Recent advancements in single-cell RNA sequencing (scRNA-seq) technology have revolutionized our ability to probe the transcriptional landscapes of cellular heterogeneous at unprecedented resolution (Lai et al., 2022; Shih et al., 2022; Tan et al., 2022). In the realm of adenomyosis research, there are few relevant studies. Some studies have discovered that there are unique cell subpopulations in adenomyosis with unique genetic and epigenetic characteristics, such as Vanin 1 (*VNN1*^+^)*EPCAM*^+^ cell subcluster, secreted frizzled-related protein 4 (*SFRP4*^+^) *IGFBP5*^hi^ natural killer T cells cells, and the cell-cell interactions occurring in the adenomyotic microenvironment, including wingless-type MMTV integration site family (WNT)/SFRP pathway, endometrial fibrosis process (Chen et al., 2022; Liu et al., 2021; Yildiz et al., 2023).

Despite these advancements, there is a notable dearth of research dedicated to identifying cellular subpopulations and delineating their roles in disease progression, particularly within various uterine regions such as the endometrium, endometrial–myometrial junction (EMJ), and myometrium. Anatomically, EMJ is a crucial component in establishing a connection between the endometrial and myometrial compartments of the uterus and its preservation is essential for maintaining proper uterine physiology (Naftalin and Jurkovic, 2009). Advanced magnetic resonance imaging reveals distinct abnormalities of EMJ in adenomyosis, rendering it an important parameter for clinical diagnosis and subtyping (Zhang et al., 2023). Nevertheless, cellular and molecular analyses of EMJ remain nascent. Historical approaches, are mainly reliant on isolated imaging or molecular techniques. The emergence of spatial transcriptomics, combined with single-cell analytics and spatial mapping, would present a robust approach to comprehensively delineate the molecular landscape of the EMJ in adenomyosis and the elucidation of the EMJ’s functional contributions to the pathogenesis of adenomyosis.

This study aims to fill this research gap by employing 10× Genomics single-cell RNA sequencing (scRNA-seq) and spatial transcriptome analysis on adenomyosis and control samples from various uterine regions. This allowed us to comprehensively characterize cellular heterogeneity, identify unique subpopulations, delineate developmental trajectories, and elucidate cell–cell communication dynamics. Differential expression analysis was implemented to uncover distinctions between adenomyotic lesions and eutopic endometrium. Through integrated analysis of the complex transcriptional landscape at single-cell resolution, this study aims to unravel novel insights into the molecular underpinnings of adenomyosis pathogenesis.

## Results

### Cellular landscape in adenomyosis explored by scRNA-seq

**Figure 1.**
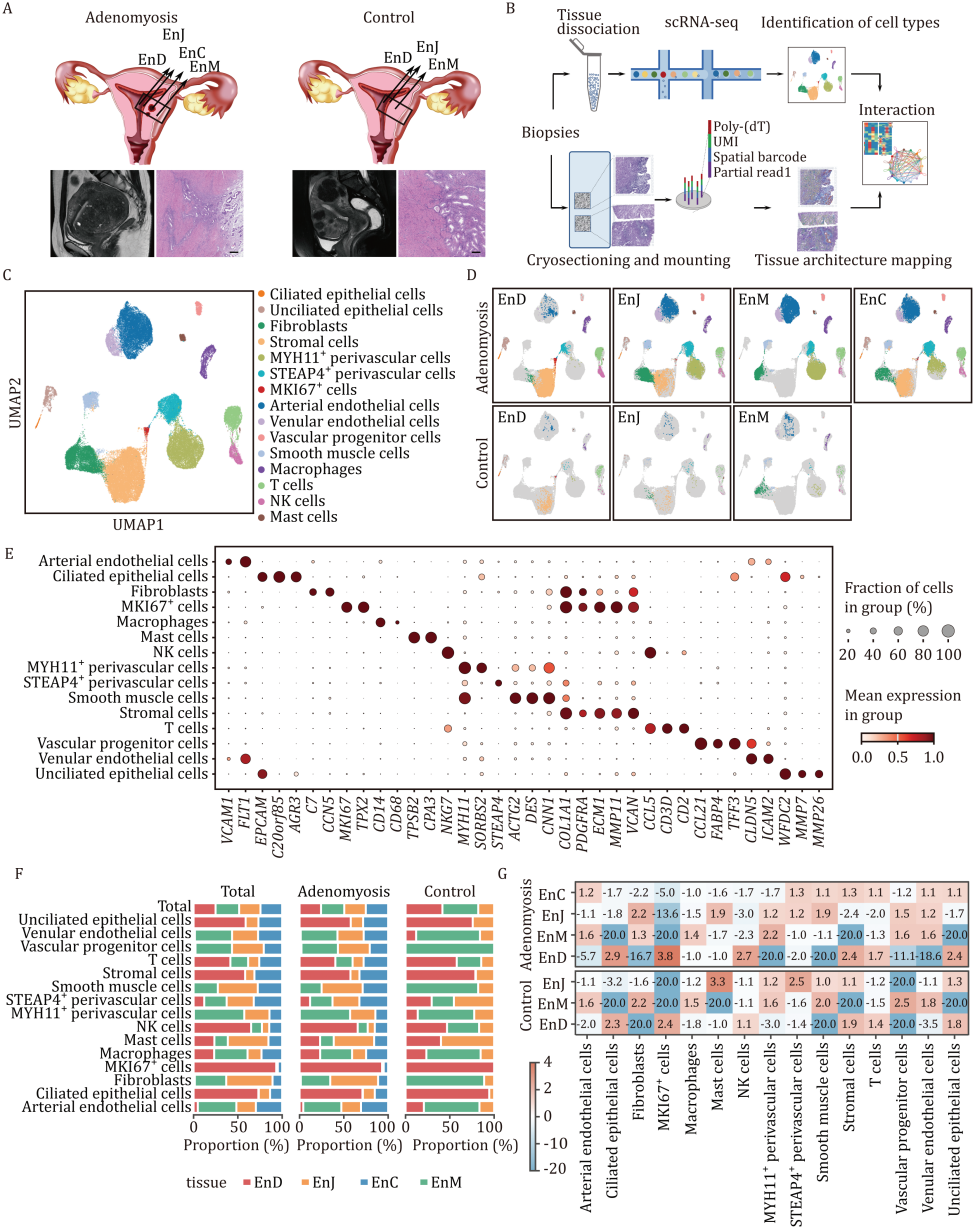
Characterization of the cell types in various regions of adenomyosis and control samples. (A) Summary of the sample origins. Schematic diagram of collected tissue biopsy samples including endometrium (EnD), endometrium–myometrial interface (EnJ), ectopic lesions (EnC), and myometrium (EnM) (top). The location of the sampling locations is based on MRI and hematoxylin and eosin staining (bottom). Uterine fibroids as controls. Scale bar: 400 μm. (B) Summary of the analysis workflow. The experimental design involved mincing the specimens, enzymatically digesting them into single-cell suspension, constructing a library, and conducting single-cell transcriptome sequencing, including 15 specimens from 4 patients (3 with adenomyosis and 1 with uterine fibroids). Additionally, a subset of the specimens underwent spatial transcriptome analysis (1 with adenomyosis). (C) The distribution of 15 main cell-type clusters in a total of 15 samples by UMAP plots. Fifteen cell types were identified with typical cell markers and visualized by UMAP plots. They included ciliated epithelial cells (*EPCAM*^+^ and *AGR3*^+^), unciliated epithelial cells (*EPCAM*^+^ and *WFDC2*^+^), stromal cells (*VCAN*^+^ and *ECM1*^+^), fibroblasts (*COL1A1*^+^), venular endothelial cells (*CLDN5*^+^), arterial endothelial cells (*FLT1*^+^), vascular progenitor cells (*CCL21*^+^ and *TFF3*^+^), smooth muscle cells (*CNN1*^+^ and *DES*^+^), MYH11^+^ and STEAP4 + perivascular cells (*MYH11*^+^ and *STEAP4*^+^, respectively), NK cells (*NKG7*^+^ and *CCL5*^+^), mast cells (*TPSB2*^+^ and *CPA3*^+^), macrophages (*CD14*^+^), T cells (*CD2*^+^), and MKI67^+^ cells (*MKI67*^+^ and *TPX2*^+^). (D) The distribution of 15 main cell type clusters in different uterine regions of adenomyosis and Ctrl samples. (E) Expression of typical marker genes of each cell type. (F) The proportion of each cell type in different uterine regions of adenomyosis and Ctrl samples by stacked bar chart. (G) Fold enrichment of each cell type in different uterine regions of adenomyosis and Ctrl samples.

We used scRNA-seq to analyze 15 tissue samples from 4 donors (3 with adenomyosis and 1 with uterine fibroids). Samples were collected from various uterine regions, including the endometrium (EnD), endometrial-myometrial junction (EnJ), ectopic lesions (EnC), and myometrium (EnM) based on the preoperative magnetic resonance image and hematoxylin-eosin staining (Fig. 1A and Table S1). Spatial transcriptomics was integrated with scRNA-seq to illuminate the cellular organization and signaling pathways (Fig. 1B). After strict quality control and standardization, 54,658 cellular transcriptomes were obtained with a median 7,731 unique transcripts and 2,142 genes per cell (Fig. S1A). Based on the expression of known markers, 15 cell types were identified with typical cell markers and visualized by uniform manifold approximation and projection (UMAP) (Figs. 1C, 1E and S1B–D). The distribution of cell types in various uterine regions and the correlation among samples were shown (Figs. 1D and S1E).

Our analysis revealed distinct cellular compositions in different uterine regions in both adenomyosis and control (Figs. 1F, 1G and S1F). As expected, epithelial and stromal cells mainly localized in EnD, EnC, and EnJ, and were not found in EnM (Fig. 1F). The majority of cell types in EnJ region of adenomyosis were fibroblasts, mast cells, smooth muscle cells, and vascular progenitor cells (Fig. 1F). Compared to controls, adenomyosis samples showed vascular progenitor cells and venular endothelial cells (EC) enrichment in EnJ (Fig. 1G). In EnC, besides stromal cells and epithelial cells, we found enrichment of multiple cell types, including six-transmembrane epithelial antigen of the prostate 4 (STEAP4^+^) perivascular cells, arterial EC and venular EC, and T cells, suggesting that abnormal vessels and immune cells may play an important role in adenomyosis development (Fig. 1G). The study identified distinct cellular compositions in various uterine regions of adenomyosis, as well as changes in the surrounding environment such as abnormal blood vessels and immune cell proliferation as important factors in its development.

### Specific gene expression of epithelial cells and featured subpopulation in adenomyosis

**Figure 2.**
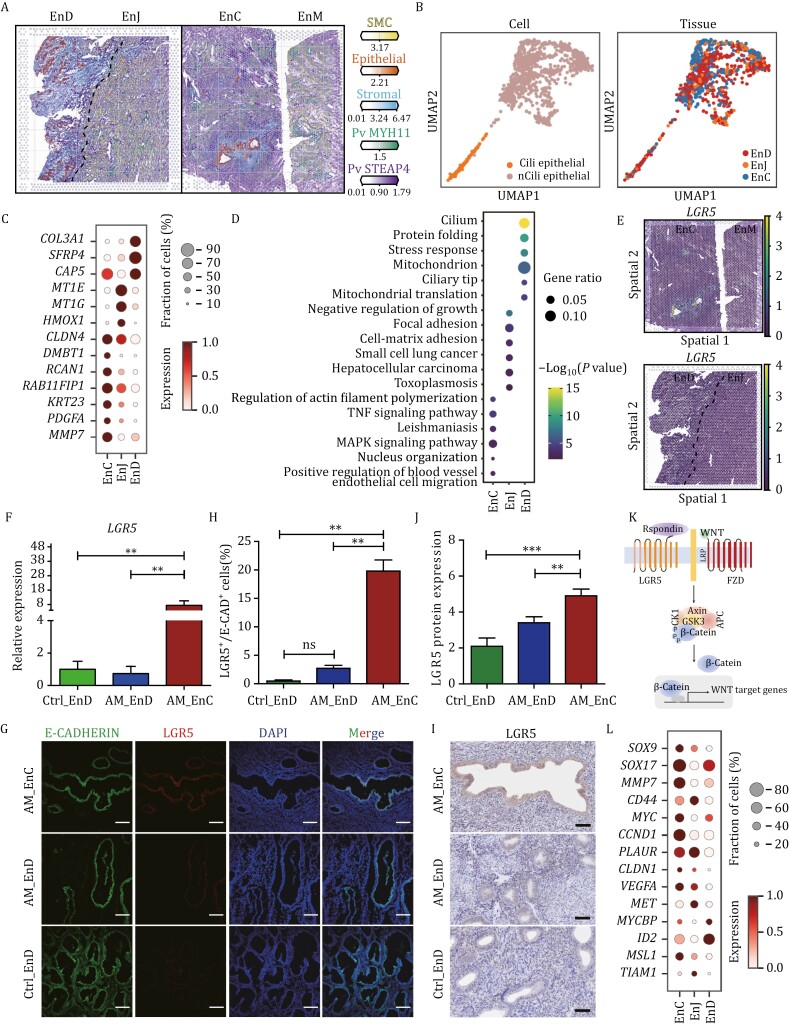
Characterization of epithelial cells in adenomyosis. (A) The overall landscape of adenomyosis was exhibited using spatial transcriptomic incorporating scRNA-seq data. Number of mRNA molecules per spot (color intensity) confidently assigned to SMCs, epithelial, stromal cells, Pv MYH11, and Pv STEAP4 in different uterine regions of adenomyosis. (B) The distribution of ciliated and unciliated epithelial cells in EnD, EnJ, and EnC in all 15 samples by UMAP. (C) The expression of differential gene expressions (DEGs) of epithelial cells in EnD, EnJ, and EnC. (D) Gene Ontology (GO) enrichment analysis of the top 100 DEGs in EnD, EnJ, and EnC. (E) Visualization of *LGR5*^+^ cells in adenomyosis samples by spatial transcriptomics. (F) mRNA expression levels of *LGR5* in epithelial cells in Ctrl_EnD, AM_EnD, and AM_EnC examined by qRT-PCR (*n* = 5 per group). Data are presented as the mean ± SEM, *P < 0.05, **P < 0.01. (G) Immunofluorescence (IF) staining for the expression of LGR5 and the epithelial marker E-CADHERIN in AM_EnC, AM_EnD, and Ctrl_EnD. Nuclei are stained with DAPI. Scale bar: 100 μm. (H) Statistical graph of the percentage of LGR5^+^ to E-CADHERIN^+^ cells in H (*n* = 3 per group). Data are presented as the mean ± SEM, *P < 0.05, **P < 0.01; ns: no significance. (I) Immunohistochemistry (IHC) staining for the expression of LGR5 in epithelial cells. Scale bar: 200 μm. (J) Protein expression of LGR5 was examined in epithelial cells in Ctrl_EnD, AM_EnD, and AM_EnC by semi-quantitative detection of immunohistochemistry (*n* = 10 per group). Data are presented as the mean ± SEM, **P < 0.01. ***P < 0.001. (K) Schematic diagram of the LGR5/WNT signaling pathway. (L) The expression of DEGs for WNT signaling pathway of epithelial cells in EnD, EnJ, and EnC.

We first showed the overall landscape of adenomyosis by using spatial transcriptomic technology incorporating scRNA-seq data. In adenomyotic lesions, epithelial cells were surrounded by stromal cells, and the ectopic lesions exhibited enrichment of STEAP4^+^ perivascular cells (PV STEAP4) compared to the eutopic endometrium, suggesting that an angiogenic microenvironment may contribute to the progression of adenomyosis (Fig. 2A).

Then, epithelial cells were divided into ciliated and unciliated cell subgroups (Fig. 2B, left). Although their distribution was consistent across different regions (EnC, EnJ, EnD) (Fig. 2B, right), their gene expressions were significantly different (Fig. 2C). The genes that were specifically expressed in EnC epithelial cells, such as matrix metallopeptidase 7 (*MMP7*), platelet derived growth factor subunit A (*PDGFA*), *KRT23* (keratin 23), *DMBT1* (deleted in malignant brain tumors 1), and *CLDN4* (claudin 4), exhibited enrichment in processes related to migration, angiogenesis, and proliferation (Fig. 2C). Gene ontology (GO) enrichment analysis showed that genes were enriched in “regulation of actin filament polymerization,” “positive regulation of blood vessel endothelial cell migration” and “tumor necrosis factor (TNF) signaling pathway” and “MAPK signaling pathway” (Fig. 2D). In contrast, genes that were specifically expressed in EnJ epithelial cells exhibited a notable upregulation of metallothionein 1E (*MT1E*), metallothionein 1G (*MT1G*), and heme oxygenase 1 (*HMOX1*), which were associated with cell growth and cell matrix adhesion (Fig. 2C and 2D). These data suggest that epithelial cells in the endometrial–myometrial junction have been altered in response to matrix remodeling and those cells in lesions exhibit migration and proliferation features.

Notably, we found leucine-rich repeat containing G protein-coupled receptor 5 (*LGR5*^*+*^) cells were significantly enriched in the epithelial cells of EnC by integrating the scRNA-seq into a spatial transcriptome (Fig. 2E). To validate the expression of *LGR5*, we performed quantitative Real-Time PCR (qRT-PCR) for epithelial cells sorted from different tissues. The results showed the mRNA expression levels of *LGR5* in EnC are significantly higher than that in EnD (Fig. 2F). Moreover, the immunofluorescent (IF) staining assay illustrates that LGR5 was specifically expressed in epithelial cells of EnC (Figs. 2G, 2H and S2A). We further verified through immunohistochemistry (IHC) staining and found that LGR5 was mainly expressed in epithelial cells of EnC of adenomyosis (Fig. 2I and 2J). LGR5 serves as a marker of adult stem cells and LGR5-expressing stem cells were reported to be essential for the development of glandular epithelial in the uterine (Seishima et al., 2019). The gene LGR5 is also recognized as a canonical target of the WNT signaling pathway. Consequently, we posited that *LGR5*^*+*^ stem/progenitor epithelial cells as a featured subpopulation in ectopic lesions and LGR5/WNT signaling pathway might contribute to adenomyosis (Fig. 2K). The expression of some genes of SRY-box transcription factor 9 (*SOX9*) (Blache et al., 2004; Liu et al., 2022), male-specific lethal 1 (*MSL1)* (Spears and Neufeld, 2011) and *MMP7* (Brabletz et al., 1999; Lv et al., 2023) related with WNT signaling pathway were significantly upregulated in EnC (Fig. 2L). Additionally, by comparing the spatial transcriptome analysis obtained from EnC and EnD, it was observed that the expression levels of *MMP7* and *CLDN4* were significantly upregulated exclusively in EnC (Fig. S2B). In conclusion, epithelial cells in adenomyosis exhibit unique gene expression profiles, with a notable presence of *LGR5*^*+*^ cells in ectopic lesions, suggesting a pivotal role in disease progression via the LGR5/WNT signaling pathway.

### Stromal cells and featured subpopulation in adenomyosis

**Figure 3.**
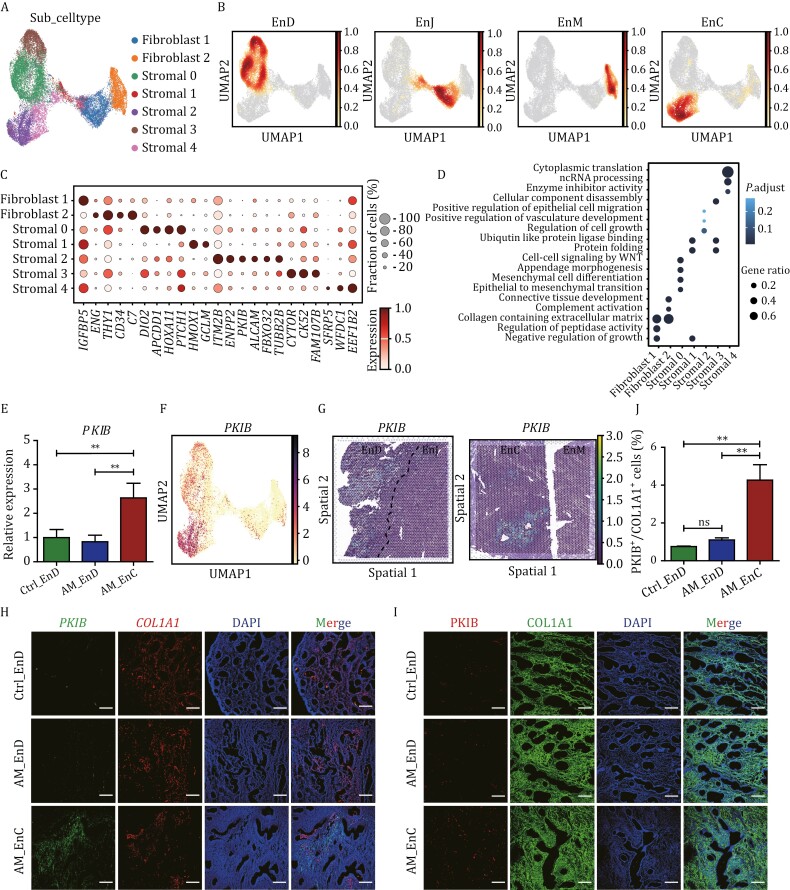
Characterization of stromal cells subpopulations in adenomyosis. (A) Stromal cells were extracted and further re-clustered and finally seven subclusters were obtained: fibroblast 1, fibroblast 2, stromal 0, stromal 1, stromal 2, stromal 3, and stromal 4. (B) The distribution of the stromal subclusters in different uterine regions of adenomyosis by UMAP plot. (C) Expression of typical marker genes of each stromal subclusters. (D) GO enrichment analysis of the top 100 DEGs in stromal subclusters. (E) mRNA expression levels of *PKIB* for stromal 2 markers in Ctrl_EnD, AM_EnD and AM_EnC examined by qRT-PCR (*n* = 6 per group). Data are presented as the mean ± SEM, *P < 0.05, **P < 0.01. (F) The expression pattern of *PKIB* in stromal 2 subclusters by UMAP plot. (G) Visualization of *PKIB* + cells in adenomyosis samples using spatial transcriptomics. (H) Fluorescence *in situ* hybridization (FISH) staining for the expression of the stromal 2 marker *PKIB* and stromal fibroblast marker *COL1A1*. Nuclei are stained with DAPI. Scale bar: 200 μm. (I) Immunofluorescence (IF) staining for the expression of the stromal 2 marker PKIB and stromal fibroblast marker COL1A1. Nuclei are stained with DAPI. Scale bar: 200 μm. (J) Statistical graph of the percentage of PKIB^+^ cells in COL1A1^+^ cells (*n* = 4 per group). Data are presented as the mean ± SEM, *P < 0.05, **P < 0.01; ns: no significance.

We re-clustered stromal cells and identified five stromal and two fibroblast subpopulations (Fig. 3A). After batch correction, different stromal subpopulation is distributed in different regions of the uterus (Fig. 3B). Stromal clusters 0 and 3 were primarily located in EnD (Fig. 3B). Stromal cluster 0 was characterized by expression of *APCDD1* (adenomatosis polyposis coli down-regulated 1), homeobox A11 (*HOXA11*) and patched (drosophila) homolog 1 (*PTCH1*), and enriched in the terms of “cell differentiation” and “WNT signaling pathway” while stromal cluster 3 enriched in ubiquitin-like protein (Ubl) conjugation and protein folding (Fig. 3C and 3D). Notably, stromal cluster 2 was unique to adenomyotic lesions (EnC) and characterized by expression of *ENPP2* (ectonucleotide pyrophosphatase/phosphodiesterase 2), *PKIB*, integral membrane protein 2B (*ITM2B)*, *ALCAM* (activated leukocyte cell adhesion molecule) and *FBXO32* (F-box protein 32) related to cell growth, cell migration and vasculature development (Figs. 3C, 3D, 3F, S3A and S3B). Stromal cluster 4 was characterized by expression of WAP four-disulfide core domain 1 (*WFDC1)*, *EEF1B2* (eukaryotic translation elongation factor 1 beta 2), and secreted frizzled-related protein (*SFRP5*) and enriched typical cytoplasmic translation (Figs. 3C, 3D and S3A). Taken together, our studies show stromal cell heterogeneity in uterine tissues.

Since stromal cluster 2 was considered to be a lesion-specific subgroup, we first validated stromal cluster 2 specific expressed genes including *ENPP2* and *PKIB* through qRT-PCR. The results demonstrated a significant up-regulation of these two genes in ectopic stromal cells compared to both control and adenomyosis patient’s stromal cells in EnD (Figs. 3E and S3C). Furthermore, *PKIB*^+^ and *ENPP2*^+^ stromal cells were uniquely distributed around epithelial cells in ectopic lesions as illustrated by spatial transcriptome (Figs. 3G and S3D). Fluorescence *in situ* hybridization (FISH) and immunofluorescence (IF) staining verified the existence and histological distribution of stromal cluster 2 (Fig. 3H–J). In summary, our results revealed a lesion-specific stromal sub-cell type in adenomyosis and found that PKIB, a gene linked to cell proliferation and invasion, effecting various cellular processes (Wan et al., 2022), was significantly increased in this cluster. The study identified five stromal and two fibroblast subpopulations in adenomyosis, with a lesion-specific stromal cluster 2 expressing proliferation and invasion-related PKIB, likely contributing to the pathogenesis of adenomyosis.

### Differentiation trajectories of stromal cells in adenomyosis and the progenitor stromal cells

**Figure 4.**
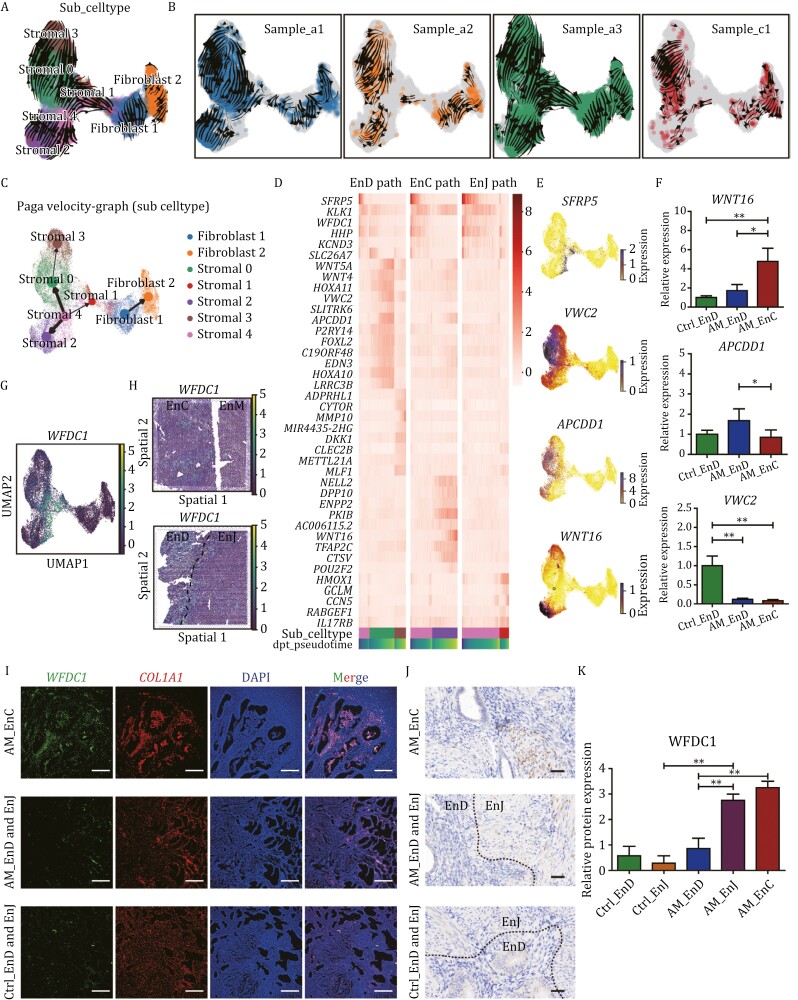
Differentiation trajectories of stromal cells in adenomyosis. (A) RNA velocity trajectory for all stromal subclusters shown by UMAP. (B) RNA velocity trajectory for the stromal subclusters of each patient sample. (C) Pseudo-time analysis of all stromal subclusters showing three trajectory fates. (D) Expression heatmap showing DEGs among three transition states, including the endometrium path (EnD path), the ectopic lesions path (EnC path) and the endometrium-myometrial junction path (EnJ path). (E) Expressions level of *SFRP5*, *VWC2*, *APCDD1*, and *WNT16* in the stromal subclusters. (F) mRNA expression levels of *WNT16*, *APCDD1*, and *VWC2* for stromal cells in Ctrl-EnD, AM-EnD, and AM-EnC examined by qRT-PCR (*n* = 6 per group). Data are presented as the mean ± SEM, *P < 0.05, **P < 0.01. (G) The expression pattern of *WFDC1* in stromal 4 subcluster by UMAP plot. (H) Visualization of *WFDC1*^+^ cells in adenomyosis samples by spatial transcriptomics. (I) FISH staining for the expression of *WFDC1* and *COL1A1*. Nuclei are stained with DAPI (blue). Scale bar: 200 μm. (J) Immunohistochemistry (IHC) staining for the expression of WFDC1 in stromal cells. Scale bar: 200 μm. (K) Protein expression of WFDC1 was examined in Ctrl_EnD and Ctrl_EnJ, AM_EnD, AM_EnC, and AM_EnJ by semi-quantitative detection of immunohistochemistry (*n* = 4 per group). Data are presented as the mean ± SEM, **P < 0.01.

To gain insight into stromal differentiation, we generated RNA velocity maps for stromal subpopulations, which predicted three developmental trajectories (Fig. 4A). Stromal cluster 4 cells were at the start site in the pseudotime trajectory, which suggests that stromal cluster 4 is the progenitor stromal cells. Stromal cluster 4 towards stromal 0, followed by stromal 3 indicated the normal endometrium path (EnD path), stromal cluster 4 towards stromal 2 indicated the ectopic lesions path (EnC path), and stromal cluster 4 towards stromal 1 indicated the endometrium-myometrial junction path (EnJ path) (Fig. 4C). It was noteworthy that the trajectory directions of the three patients with adenomyosis remained completely consistent (Fig. 4B).

We next mapped differentially expressed genes (DEGs) between the cell subclusters and along the pseudotime trajectories (Fig. 4D). A notable upregulation of *SFRP5* was detected in progenitor stromal cells, primarily localized within stromal cluster 4 (Fig. 4D and 4E). Given its role as a suppressor of the WNT signaling pathway, this gene encoding a protein hormone may potentially modulate mechanisms in the pathogenesis of adenomyosis. In EnD path, gene related to WNT signaling pathway (such as *APCDD1*, Von Willebrand factor C domain containing 2 (VWC2), wingless-type MMTV integration site family, member 5A (*WNT5A*), wingless-type MMTV integration site family, member 4 (*WNT4*)) and uterus development genes (including homeobox A10 (*HOXA10*), *HOXA11*, forkhead box L2 (*FOXL2)*) were up-regulated. While in the EnC path, the expression level of *ENPP2*, *PKIB*, wingless-type MMTV integration site family, member 16 (*WNT16*), and neural EGFL like 2 (*NELL2)*, were increased (Fig. 4D and 4E). To validate the genes in the path, the expression level of *WNT16*, *APCDD1*, *VWC2* was further verified by qRT-PCR (Fig. 4F).

To further identify signature genes of stromal cluster 4, we found that *WFDC1* was the top marker gene, which is associated with inflammation, repair, and cell migration and is mainly expressed in stromal 4 cluster (Figs. 3C and 4G). *WFDC1*^+^ cells were mainly enriched in EnD, EnJ, and EnC of adenomyosis by spatial transcriptome (Fig. 4H). Therefore, we further validated through FISH and IHC staining and found that WFDC1 was mainly expressed in the EnC and EnJ regions of adenomyosis (Figs. 4I–K and S4A). In conclusion, stromal progenitor cells differentiate towards lesional cells or normal endometrium via distinct trajectories in adenomyosis, providing *WFDC1*^+^ stromal progenitor cells may serve as precursor cells of lesion-specific stromal cluster and play an important role in the development of adenomyosis.

### Endothelial cell diversity and angiogenesis

**Figure 5.**
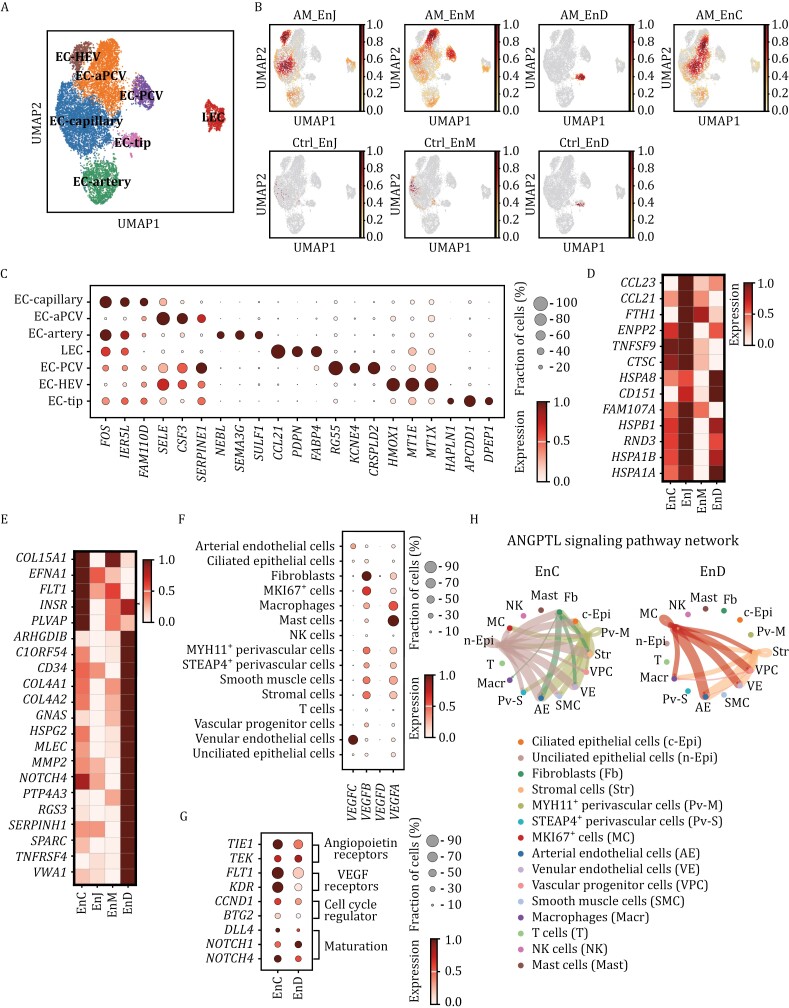
Characterization of EC in adenomyosis. (A) EC were extracted and further re-clustered and finally seven subclusters were obtained: lymphatic EC (LEC), high endothelial venule (EC-HEV), capillary (EC-capillary), post-capillary venous (EC-PCV), activated PCV (EC-aPCV), EC-tip and arterial (EC-artery). (B) The distribution of the endothelial subclusters in different uterine regions of adenomyosis and control samples by UMAP plot. (C) Expression of typical marker genes of each endothelial subclusters. (D) The expression of upregulated immune-related and chemokines genes in different uterine regions of adenomyosis by heatmap. (E) The expression of upregulated angiogenesis-associated genes in different uterine regions of adenomyosis by heatmap. (F) The expression signaling patterns of *VEGF* in various cell types by Dot plot. (G) The expression of significant DEGs of EC-tip endothelial cell subsets in EnC and EnD of adenomyosis. (H) Ligand-receptor pairs network of ANGPTL signaling pathways for intercellular communication in EnC and EnD.

Endothelial cells（EC）were significantly increased in EnC, which indicates that angiogenesis occurs in adenomyotic lesions (Fig. 1G). We identified seven of EC subsets with varying distributions in adenomyotic tissue (Figs. 5A, 5B and S5A). For example, EC-capillary clusters were mainly distributed in EnC, EnJ, and EnM, which was similar in the control group and the adenomyosis group. Furthermore, gene expression analysis revealed differentially upregulated immune-related and angiogenesis-related genes in different adenomyotic regions (Fig. 5C and 5E). In EnJ, there was an increased expression of genes linked to chemokines (*CCL23* (C-C motif chemokine ligand 23), *CCL21* (C-C motif chemokine ligand 21)) and immune responses (TNFSF9 (tumor necrosis factor superfamily member 9), *CTSC* (cathepsin C gene)) (Fig. 5D). In EnD, angiogenesis-associated genes such as *ARHGDIB* (Rho GDP dissociation inhibitor beta) and GNAS (guanine nucleotide-binding protein alpha stimulating) were predominantly up-regulated (Fig. 5E). Distribution of plasmalemma vesicle-associated protein positive (*PLVAP*^+^) cells could influence the permeability of EC and regulate vascular permeability (Denzer et al., 2023) were observed around the lesion by spatial transcriptome (Fig. S5B). Furthermore, our study also revealed distinct vascular endothelial growth factor (VEGF) and ANGPT signaling patterns in various cell types, indicating a complex angiogenic environment (Figs. 5F and S5C).

Angiogenic signaling and neovasculature were altered in endothelial cell subsets. EC-tip, responding to angiogenic signals, migrates and proliferates to form new vascular structures and differentiate into capillary, arterial, and venous endothelial subtypes (Lee et al., 2021). The up-regulation of ANGPT (angiopoietin) and VEGF receptors tyrosine kinase with immunoglobulin-like and EGF like domains 1 (*TIE1*) (La Porta et al., 2018), Fms related tyrosine kinase 1 (*FLT1*) (*VEGFR1*) (Stefater et al., 2011, 2013) and kinase insert domain receptor (*KDR*) (*VEGFR2*) (Das et al., 2022) in EnC of EC-tip cells indicates active angiogenesis (Huang et al., 2010; Tan et al., 2022) (Fig. 5G and S5D). Additionally, the activation of delta-like ligand 4- Notch (drosophila) homolog (NOTCH) signaling suggests the maturation of EC-tip cells (Fig. 5G). Moreover, the ANGPT-TIE axis is identified as crucial for angiogenesis, and extensive intercellular communication of angiopoietin-like (ANGPTL), ANGPT, and VEGF signals is observed in EnC of adenomyosis (Figs. 5H, S5E and S5F). In EnC, ANGPTL signals were released from nCili epithelial cells, myosin heavy chain 11 (*MYH11*^+^) perivascular cells, and fibroblasts and targeted to endothelial cells, stromal cells and *STEAP4*^*+*^ perivascular cells. Overall, endothelial cell heterogeneity and differential angiogenesis-related signaling were found across adenomyotic regions. Aberrant vascular permeability and angiogenesis likely contribute to symptoms like heavy menstrual bleeding.

### Aberrant cell connections in adenomyosis

**Figure 6.**
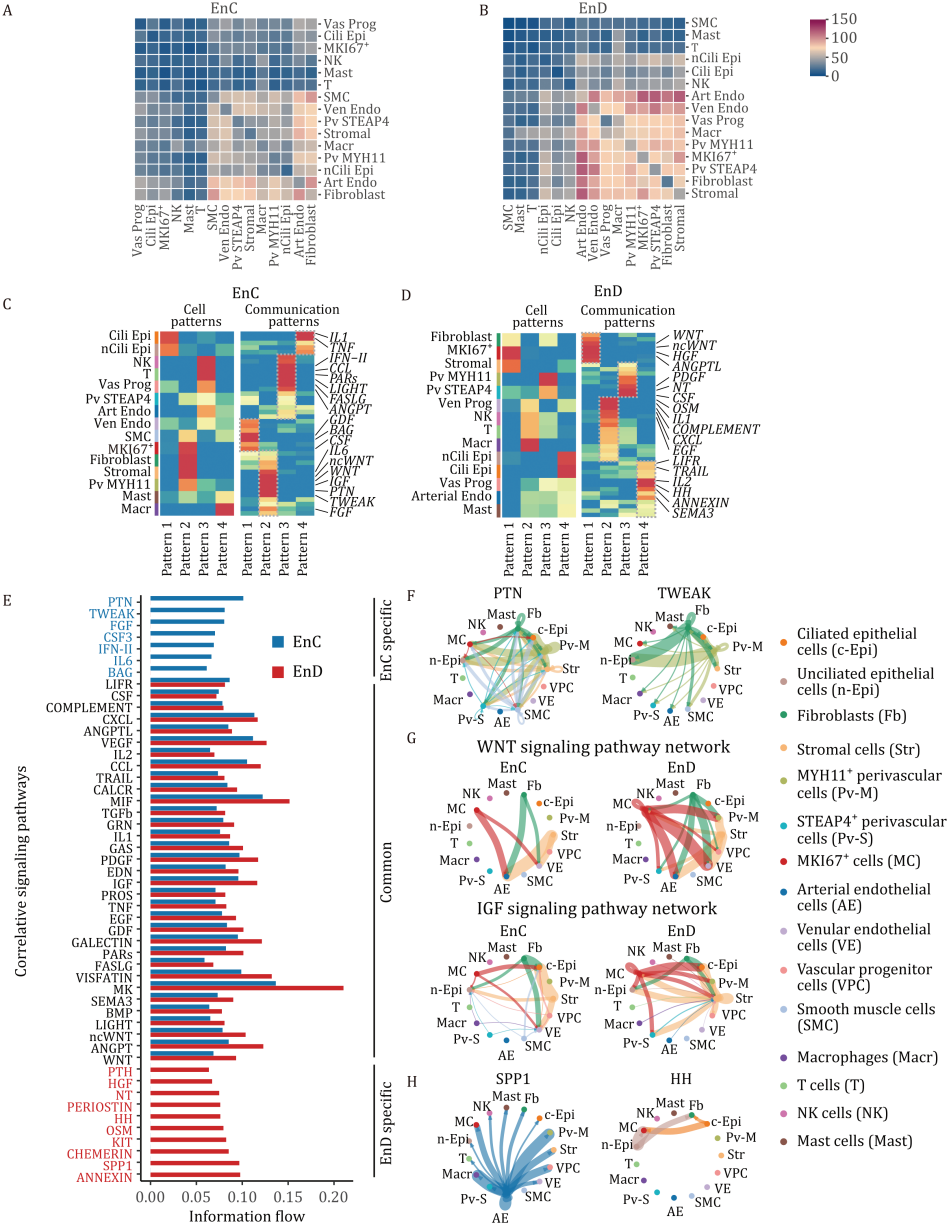
Cell-to-cell crosstalk in EnD and EnC of adenomyosis. (A) The number of connections between different cell types in EnC of adenomyosis by CellphoneDB: vascular progenitor cells (Vas Prog), ciliated epithelial cells (Cili Epi), MKI67^+^ cells (MKI67^+^), NK cells (NK), mast cells (Mast), T cells (T), smooth muscle cells (SMC), venular EC (Ven Endo), STEAP4^+^ perivascular cells (Pv STEAP4), stromal cells (stromal), macrophages (Macr), MYH11^+^ perivascular cells (Pv MYH11), unciliated epithelial cells (nCili Epi), arterial EC (Art Endo), fibroblasts (fibroblast). (B) The number of connections between different cell types in EnD of adenomyosis by CellphoneDB. (C) Heatmap of signal patterns of different cell types in EnC of adenomyosis shown by CellChat. (D) Heatmap of signal patterns of different cell types in EnD of adenomyosis shown by CellChat. (E) Bar graph shows the correlation of signaling pathway in EnC and EnD. (F) Circle plot showing the inferred PTN and TWEAK signaling networks between different cell types in EnC. (G) Circle plot showing the inferred WNT and IGF signaling networks between different cell types in both EnC and EnD. (H) Circle plot showing the inferred SPP1 and HH signaling networks between different cell types in EnD.

Finally, we used CellPhoneDB and CellChat to analyze cell-to-cell interactions in adenomyotic ectopic and eutopic endometrial tissues. In EnC, interactions between stromal cells, fibroblasts, and EC were reduced, while smooth muscle cells (SMCs) showed increased interactions with other cell types (Fig. 6A and 6B).

Different signaling pathways pattern of cell communications were enriched in EnD and EnC of adenomyosis (Fig. 6C and 6D). In EnC, pleiotrophin (PTN) and tumor necrosis factor-like weak inducer of apoptosis (TWEAK) were the predominant output signals, mainly secreted by fibroblasts and *MYH11*^+^ perivascular cells (Pv MYH11) (Fig. 6E and 6F). Moreover, other aberrant signaling pathways, such as fibroblast growth factor (FGF), interleukin 6 (IL6), and Bcl2-associated athanogene (BAG), were observed in EnC (Fig. S6A). These signaling pathways were linked to cell proliferation, angiogenesis and immune responses, and promoted the ectopic lesions progression. In contrast, EnD showed enrichment in secreted phosphoprotein 1 (SPP1) and hedgehog (HH) pathways, associated with endometrial receptivity and stromal cell activities (Fig. 6H) (Altmäe et al., 2017). Additionally, other cellular interaction signaling pathways were up-regulated in EnD, particularly in interactions involving macrophages and other cellular phenotypes (Fig. S6C).

EnC and EnD exhibited differential expressions in crucial signaling pathways like WNTs, insulin-like growth factors (IGFs), and tumor necrosis factor (TNF) (Figs. 6G and S6B). WNTs and IGFs, secreted by marker of proliferation Ki-67 (MKI67^+^) cells, fibroblast, and stromal in EnD, were diminished in EnC. The communications involved immune cells, especially macrophages and T cells, were changed as well, inducing TNF, colony stimulating factor (CSF), leukemia inhibitory factor receptor (LIFR), and Interleukin-2 (IL2) signaling pathway (Fig. S6B). Taken together, our findings showed altered communication patterns in adenomyotic tissues. Changes in signaling pathways, including those related to cell proliferation, angiogenesis, and immune responses, were observed. This points to a dynamically altered microenvironment in adenomyosis, contributing to its complex pathophysiology.

## Discussion

Adenomyosis represents an incompletely understood gynecological disorder. To address these gaps, in this study, we performed single-cell RNA sequencing and spatial transcriptomic analysis on adenomyosis and control samples from covering all important regions of the entire uterus. This allowed us to comprehensively characterize cellular heterogeneity in adenomyosis and elucidate regional molecular heterogeneity, notably within the pivotal endometrial–myometrial junction. We identified unique epithelial and stromal subpopulations specific to adenomyotic lesions, revealed a complex angiogenic microenvironment, and delineated altered intercellular communications. The identification of discrete cell populations, aberrant signaling programs, and regional distinctions advance our understanding of the intricate molecular landscape in adenomyosis pathogenesis.

We focus on comparing the cellular characteristics of eutopic and ectopic endometrium locations and seek targeted treatment strategies for ectopic lesions. This study identified unique epithelial (*LGR5*^+^) and stromal (*PKIB*^+^) subpopulations specific to adenomyotic lesions through single-cell analysis. Additionally, a putative stromal progenitor (*WFDC1*^+^) was revealed. PKIB regulates cell proliferation and invasion patterns in various types of cancer (Zhang et al., 2017). The presence of invasive *PKIB*^+^ stromal cells implicated abnormalities in stromal cell signaling and activities in driving lesion formation, consistent with the “invagination” theory whereby aberrant stromal-epithelial interactions facilitate endometrial invasion into the myometrium. However, LGR5 has been reported as a marker of adult stem cells (Barker et al., 2007; Leung et al., 2018; Zhang et al., 2018). In mice, *LGR5*^+^ cells are essential for uterine gland development (Seishima et al., 2019). In healthy human premenopausal endometrium, LGR5 is mainly expressed in the luminal epithelial cells (de Visser et al., 2012; Tempest et al., 2018). Meanwhile, the identification of a *WFDC1*^+^ stromal progenitor population, potentially serving as precursors to lesion-specific stromal cells, provides evidence for the involvement of stem/progenitor cells in adenomyosis pathogenesis, aligning with the “metaplasia” theory (Zhu et al., 2021). It may reshape our understanding of adenomyosis pathology that the identification of unique epithelial, invasive stromal, and progenitor stromal subpopulations supports the complex interplay of both the “invagination” and “metaplasia” theories in adenomyosis origin.

The endometrial-myometrial junctional zone has become increasingly prominent, as aberrations herein can lead to adenomyosis and infertility (Pados et al., 2023). Prior evidence indicates this zone may harbor stem/progenitor cells that contribute to endometrial regeneration during menstruation, aligning with our observation of *WFDC1*^+^ stromal cells (Kobayashi et al., 2020). These highly ribosomal progenitor-like cells likely represent a stem cell population, as ribosomal suppression and lineage-specific upregulation enable commitment (Athanasiadis et al., 2017; Signer et al., 2014). Moreover, we observed vascular progenitor cells and mast cells enriched in EnJ of adenomyosis, which is consistent with the literature showing the presence of perivascular inflammatory cell infiltration in adenomyosis (Bourdon et al., 2021). *MT1E*, *MT1G*, and *HMOX1*, associated with cell growth and matrix adhesion were upregulated in epithelial cells of EnJ. Thus, aberrant remodeling and inflammation may synergize at the junctional zone, enabling endometrial invasion into the myometrium. Elucidating junctional zone alterations and resident stem-like populations provide critical insight into early adenomyosis pathogenesis.

Angiogenesis holds particular importance in adenomyosis, with our data revealing altered signaling and neovasculature in some pivotal endothelial cell subsets (Harmsen et al., 2022). The pivotal ANGPT pathway showed dysregulation, aligning with other angiogenic studies (Huang et al., 2010). Aberrant intercellular communications between epithelial, stromal, perivascular cells, endothelial, and immune cells were altered in adenomyosis lesions. Specific enrichment of PTN and TWEAK pathways occurred, both implicated in proliferation, migration, differentiation, and angiogenesis (Shi et al., 2017; Wang et al., 2022). Crucial differences in WNT, IGF, and TNF signaling arose between lesions and eutopic endometrium. Ultimately, adenomyosis demonstrates strikingly complex and dynamic cellular signaling patterns. Elucidating these angiogenic, inflammatory and signaling aberrancies are key to deciphering cryptic disease mechanisms in this heterogeneous disorder.

Our study provides seminal insights into adenomyosis pathology. First, we establish a high-resolution single-cell map, elucidating junctional zone heterogeneity for the first time. Second, this study identified unique epithelial (*LGR5*^+^) and stromal (*PKIB*^+^) subpopulations specific to adenomyotic lesions through single-cell analysis. LGR5 has been reported as a marker of adult stem cells, and the identification of a *WFDC1*^+^ stromal progenitor population, potentially served as precursors to lesion-specific stromal cells. We provided insights into the interplay between the metaplasia and invagination theories. Third, the defined populations offer targets for precision diagnostics and therapeutics. Targeting the invasive *PKIB*^+^ stromal or progenitor *LGR5*^+^ epithelial cells may enable the development of targeted therapeutics. The angiogenic and immune signaling aberrancies highlight the potential for novel anti-angiogenic and immunomodulatory therapies. Ultimately, this integrated single-cell analysis fundamentally advances and reshapes the understanding of adenomyosis.

In conclusion, this study elucidates the complex single-cell combined spatial transcriptional landscape of adenomyosis. The study provides insights into the interplay between the metaplasia and invagination theories. Our study uncovers molecular alterations of adenomyosis, offering avenues for precise diagnosis and treatment, and highlights the need for clinical validation of these promising findings.

## Limitations of the study

While our study is informative, it has limitations that must be addressed in future studies. The sample size was limited, and the functional roles of the identified cell subpopulations and signaling pathways in adenomyosis are yet to be fully understood. Future studies should aim to validate these findings in larger cohorts and employ functional assays to confirm the roles of these cells and pathways in the disease’s progression. Additionally, the clinical implications of our findings, such as their relationship to symptoms like dysmenorrhea and heavy menstrual bleeding, require further exploration.

## Materials and methods

### Human subjects

Three adenomyosis patients and 1 patient with uterine fibroids were in the proliferative phase of the menstrual cycle after hysterectomy (see Methods and Table S1) at Jiaxing University Affiliated Maternity and Child Hospital. These patients routinely underwent hysteroscopy and biopsy. All sampling and experimental procedures were approved by the Scientific Research Ethics Committee of the Jiaxing University Affiliated Maternity and Child Hospital (No. 2021-65), and informed consent was obtained from each participant.

### Sample collection and preparation

To explore the cellular landscape in adenomyosis, we performed scRNA-seq (10× Genomics) for 15 specimens collected from the uteri of four donors who were in the proliferative phase of the menstrual cycle after hysterectomy (see Methods and Table S1). Samples from three adenomyosis patients were collected from various uterine regions, including the endometrium (EnD), endometrial-myometrial junction (EnJ), ectopic lesions (EnC), and myometrium (EnM) by an experienced chief gynecologist. Biopsies of EnD, EnJ, and EnM were collected from one patient with uterine fibroids as control. The location of biopsies was based on the preoperative magnetic resonance image, and HE staining was performed to further verify the histological characteristics and the accuracy of the biopsies site. To understand the spatial organization and potential cell signaling pathways responsible for adenomyosis pathology, we simultaneously performed spatial sc-RNAseq on one of the adenomyosis samples derived from single-cell transcriptome analysis using two tissue sections: one from the junction of endometrium and myometrium (EnD and EnJ regions), and another from the adenomyosis lesion and adjacent myometrium (EnC and EnM regions).

### scRNA-seq data processing

We employed 10× Genomics scRNA-seq technology to capture and sequence individual cells from the 15 tissue samples. The sequence data were mapped to the hg38 human genome to perform quality control and the read counting of Ensemble genes using Cell Ranger (v.7.1.0) with default parameters. The gene-cell sparse matrix was generated for each sample by Cell Ranger software.

All 15 samples from patients and control were integrated and analyzed following the standard pipeline of the Scanpy package (v1.9.3). In brief, we concatenated the count matrices from all the samples and merged the matrix. We only keep good quality cells that meet the following criteria: (i) cells with between 700 and 5,000 genes expressed; (ii) cells with UMI count less than 20,000; and (iii) cells with mitochondrial gene expression percentages fewer than 15. Genes expressed in less than 20 cells were removed, but we did not set this number too high to avoid restricting rare cell type detection. To detect potential doublets, the Scrublet (v0.2.1) pipeline was performed on each sample by setting parameters ‘n_prin_comps=30’, ‘expected_doublet_rate =0.06’, and ‘sim_doublet_rate =20’. A total of 9,847 cells with doublet scores greater than the threshold were identified as doublets and excluded from subsequent analysis. Overall, 54,658 single cells with mean 2,142 genes per cell genes were retained. Next, the filtered gene expression matrix was normalized and log-transformed using sc.pp.normalize_total and sc.pp.log1p. Highly variable genes were calculated by sc.pl.highly_variable_genes and used to perform principal component analysis (PCA) with n_comps = 50. Then the harmony algorithm was used to perform batch correction to integrate different samples using default parameters. The batch-corrected PCs were used for further analysis such as the nearest-neighbor graphs. Finally, the neighborhood graph computed from the pp.ngighbors function was utilized for unsupervised clustering performed by the Leiden algorithm.

To minimize the effect of cell cycle heterogeneity, we first downloaded the cell cycle gene list, and then the cell cycle scores for every single cell were calculated with the score_genes_cell_cycle function in Scanpy. We found none of the 20 first PCs had cell cycle genes within the top 20 positive and negative genes, and so cell cycle regression was not performed.

The clusters were identified as different major cell lineages based on the average gene expression of well-known markers, including ciliated epithelial cells (*AGR3*^+^ and *EPCAM*^+^), unciliated epithelial cells (*WFDC2*^+^), stromal cells (*VCAN*^+^ and *ECM1*^+^), fibroblasts (*COL1A1*^+^), venular EC (*CLDN5*^+^), arterial EC (*FLT1*^+^), vascular progenitor cells (*CCL21*^+^ and *TFF3*^+^), smooth muscle cells (*CNN1*^+^ and *DES*^+^), *MYH11*^+^ and *STEAP4*^+^ perivascular (Pv) cells (*MYH11*^+^ and *STEAP4*^+^, respectively), NK cells (*NKG7*^+^ and *CCL5*+), mast cells (*TPSB2*^+^ and *CPA3*^+^), macrophages (*CD14*^+^), T cells (*CD2*^+^), and MKI67^+^ cells (*MKI67*^+^ and *TPX2*^+^). Sub-cluster annotation of cell lineages refers to the specific genes listed in Dataset EV3.Repeating the process (normalization, dimensionality reduction and clustering). Sub-clusters for stromal cells and EC were further identified and annotated as different specific cell subtypes based on the average expression of respective gene sets in each major cell type.

### Spatial transcriptomics analysis

Space Ranger (version 1.3.1) software from 10× genomics was used to perform process, alignment, and barcode/UMI counting against the human hg38 reference genome for each spot on the Visum spatial transcriptomic array. To spatially map cell types defined by scRNA-seq analysis within Visium spatial transcriptomics data, we employed cell2location. In essence, cell2location uses a spatially resolved approach to decompose multi-cell spatial transcriptomics data into estimates of cell-type abundance. Initially, the models derive expression signatures of cell types by computing the average expression counts of each gene in each cell type from the raw count scRNA-seq data, selecting genes expressed in at least three cells. Subsequently, to obtain the location of cell types, the model performs a hierarchical non-negative decomposition of the gene expression profiles at spatial locations (spots with multiple cells) to derive reference signatures. Each Visium section was analyzed separately with default values, except train_args = ‘n_iter’:30,000;posterior_args = ‘n_samples’:1,000;model_kwargs = ‘cell_number_prior’:{‘cells_per_spot’:8,‘factors_per_spot’: 4}; and ‘gene_level_prior’: {‘mean’: 1/2, ‘sd’: 1/4, ‘mean_var_ratio’: 1}.

### Identification of DEGs

The DEGs among the clusters were identified using the tl.rank_genes_groups function in Scanpy. Genes with FDR-corrected *P*-value < 0.05 and log fold change > 1 were considered significantly high in that cluster or sub-cluster. The enriched GO terms of biological processes for the DEGs were identified by the enrich function of R package Clusterprofiler (v4.0.1).

### Cell-type proportion and enrichment analysis

For each sample, cell-type proportions were calculated by dividing the number of cells in a cluster by the total number of cells in the sample. Similarly, for each tissue, cell-type proportions were calculated by dividing the number of cells in a cluster by the total number of cells in the tissue. Fold enrichment and depletion of each cell type across different clusters was calculated as the log2 ratio of the observed cell proportion over expected cell proportion across different tissues. The expected cell proportions were calculated as the number of total cells divided by the number of each cell cluster. The observed cell proportion was calculated as the number of cells in a given tissue divided by the number of each cell cluster in that tissue.

### RNA velocity analysis

To understand the developmental trajectories of stromal cells in adenomyosis, RNA velocity analysis was performed. This helped in identifying the origin and potential fate of various stromal subpopulations. We ran the run_10 × command of velocyto package to process the Cell Ranger aligned bam files, the count matrix made of spliced and unspliced read counts was outputted to loom file. Next, the merged spliced/unspliced counts object was further merged with the AnnData object using the scv.utils.merge function in the scVelo (v0.2.5) package. For the genes used for velocity calculation, we used the default parameter to calculate the top 2,000 highly variable genes. The stochastic model was selected for velocity estimation by running the scv.tl.velocity. The velocity graph was computed by scv.tl.velocity_graph. As a result, transition probabilities were estimated to form a velocity graph. We embedded the resulting velocities on the low dimensional space using the velocity_embedding_stream function. Finally, we used scv.pl.paga to abstract information from RNA velocity.

To identify genes that may explain the trajectory, we test which genes have significantly differential sub-cluster specific velocity expression by scv.tl.rank_velocity_genes with threshold min_corr = .3. Gene expression and annotation changes along paths in the abstracted graph were plotted by using sc.pl.paga_path.

### Cell–cell communication analysis

To study the cell–cell communication between different cluster or sub-clusters, CellChat (v1.6.1) was applied to infer the ligand–receptor pairs between cell types. In brief, gene expression data of cells and assigned cell types were used as input for CellChat. First, overexpressed ligands or receptors in one cell group were identified using identity overexpressed genes function, and then gene expression data were projected onto the protein–protein interaction network. The used human database is ‘secreted signaling’. To obtain strong signaling pathways, we set the parameter of “min.cells” to be 10 for the filter Communication function. Validation Experiments

### Quantitative reverse transcription PCR (qRT-PCR)

Total RNA was extracted using TRNzol Universal reagent (Tiangen) and reverse transcribed using the 5× all-in-one MasterMix (Abm G490). Quantitative reverse-transcription PCR was performed with SYBR Premix Ex Taq (Takara) and the ABI7500 Fast Real-time PCR system (Applied Biosystems). The reactions were performed in triplicate using 1/10 concentration of the cDNA obtained as described above. Relative mRNA expression is normalized to GAPDH as an endogenous control using the ΔΔCT or ΔCT method. The primer sequences used in this study are listed in Table S3.

### Frozen section immunofluorescence (IF) staining, and RNA fluorescence *in situ* hybridization (FISH)

Endometrial tissues were fixed by 4% paraformaldehyde at 4°C overnight, and then washed three times with PBS, dehydrated by 30% sucrose for 1 h, embedded in OCT, and transferred to −80°C refrigerator overnight. Embedded tissues were sectioned by Leica frozen slicer at a thickness of 15 μm, the slices were baked on a heating plate for 40 min at 45°C. Before staining, the slices were washed three times with PBS for clearing OCT, and incubated with 0.3 mol/L glycine to clear the aldehyde group. After permeabilizing and blocking with 0.5% Triton X-100 in 3% BSA/PBS solution for 4 h at room temperature, the sections were incubated with primary antibodies at 4°C overnight and then washed three times with 0.05% Tween-20. The following primary antibodies were used: mouse anti-LGR5 (Abcam, ab273092, 1:100), rabbit anti-E-cadherin (Cell signaling, 3195T, 1:1,600), rabbit anti-COL1A1 (Cell signaling, 72026, 1:100), mouse anti-α-SMA (Abcam. ab7817.1:200), mouse anti-Desmin (Invitrogen, MA5-13259, 1:50), and rabbit anti-PKIB (Abcam, ab196689, 1:300). The secondary antibodies were incubated at room temperature for 2–3 h, and the slides were washed three times with 0.05% Tween-20.

For RNA FISH to detect *WFDC1*, *PKIB,* and *COL1A1*, the probes were designed by Servicebio Technology Co., Ltd., Wuhan, China. All operations were carried out according to the manufacturer’s instructions. Briefly, the sections were washed three times with PBS, permeabilized with 1× proteinase K at 40°C for 20 min, and washed three times by PBS again. Prehybridization was performed by incubating the sections at 40°C for 40 min in hybridization solution. After incubating with pre-heated hybridization probe mix1 for overnight at 40°C, the slides were washed with 2× SSC, 1× SSC, 0.5× SSC, 0.1× SSC at 40°C for 15 min each time. Afterwards, the probes mix2 and fluorescent probes were incubated step by step and washed as described above. Samples were mounted in 50% glycerin and images were acquired by a confocal laser scanning microscope (Olympus FV3000).

### Immunohistochemical analysis

Sections from adenomyosis specimens underwent immunohistochemical assays as outlined in the prior methodology (Xu et al., 2017). Two independent investigators assessed the results using a semi-quantitative scale. WFDC1 expression grading amalgamated percentage and intensity scores. Percentage scores were assigned based on the proportion of positively stained cells: 0 for none, 1 for up to 25%, 2 for > 25% to 50%, and 3 for > 50%. Intensity scores were categorized as 0 for no staining, 1 for weak, 2 for moderate, and 3 for high staining (Zhang et al., 2010).

### Statistical analysis

SPSS 19.0 and Graph Pad Prism 5 were used for statistical analysis. Data are shown as mean ± SEM. *P* values were calculated using the two-tailed Student’s *t*-test or Mann–Whitney *U* test for two groups and a one-way ANOVA for more than two groups. A statistical difference was considered significant at *P* < 0.05 (*), very significant at *P* < 0.01 (**), and not significant at ns.

## Supplementary data

The online version contains supplementary material available at https://doi.org/10.1093/procel/pwae012.

pwae012_suppl_Supplementary_Tables_S1-S3_Figures_S1-S6

## Data Availability

The raw sequence data reported in this paper have been deposited in the Genome Sequence Archive in National Genomics Data Center, China National Center for Bioinformation/Beijing Institute of Genomics, Chinese Academy of Sciences (GSA-Human: HRA005777) that are publicly accessible at their website. This paper does not report custom code.

## Code availability

Any additional information required to re-analyze the data reported in this work paper is available from the lead contact upon request.
