# Supplementary material for: Comprehensive transcriptional atlas of human adenomyosis deciphered by the integration of single-cell RNA-sequencing and spatial transcriptomics
Source: Protein Cell. 2024 Mar 15;15(7):530–46. doi: 10.1093/procel/pwae012 (PMC11214835; doi:10.1093/procel/pwae012)
Supplement: pwae012_suppl_Supplementary_Tables_S1-S3_Figures_S1-S6 [file pwae012_suppl_supplementary_tables_s1-s3_figures_s1-s6.pdf]

SI Appendix

Comprehensive transcriptional atlas of human adenomyosis deciphered by the integration of single-cell RNA-sequencing and spatial transcriptomics

Tao Chen[1,2,8], Yiliang Xu[3,8], Xiaocui Xu[2,8], Jianzhang Wang[4,8], Zhiruo Qiu[5], Yayuan Yu[1], Xiaohong Jiang[1], Wanqi Shao[6], Dandan Bai[2], Mingzhu Wang[2], Shuyan Mei[5], Tao Cheng[5], Li Wu[7,*], Shaorong Gao[2,*], Xuan Che[1,5,9,*]

This word file includes:

Figure S1 to S6

Table S1 to S3

Figure S1

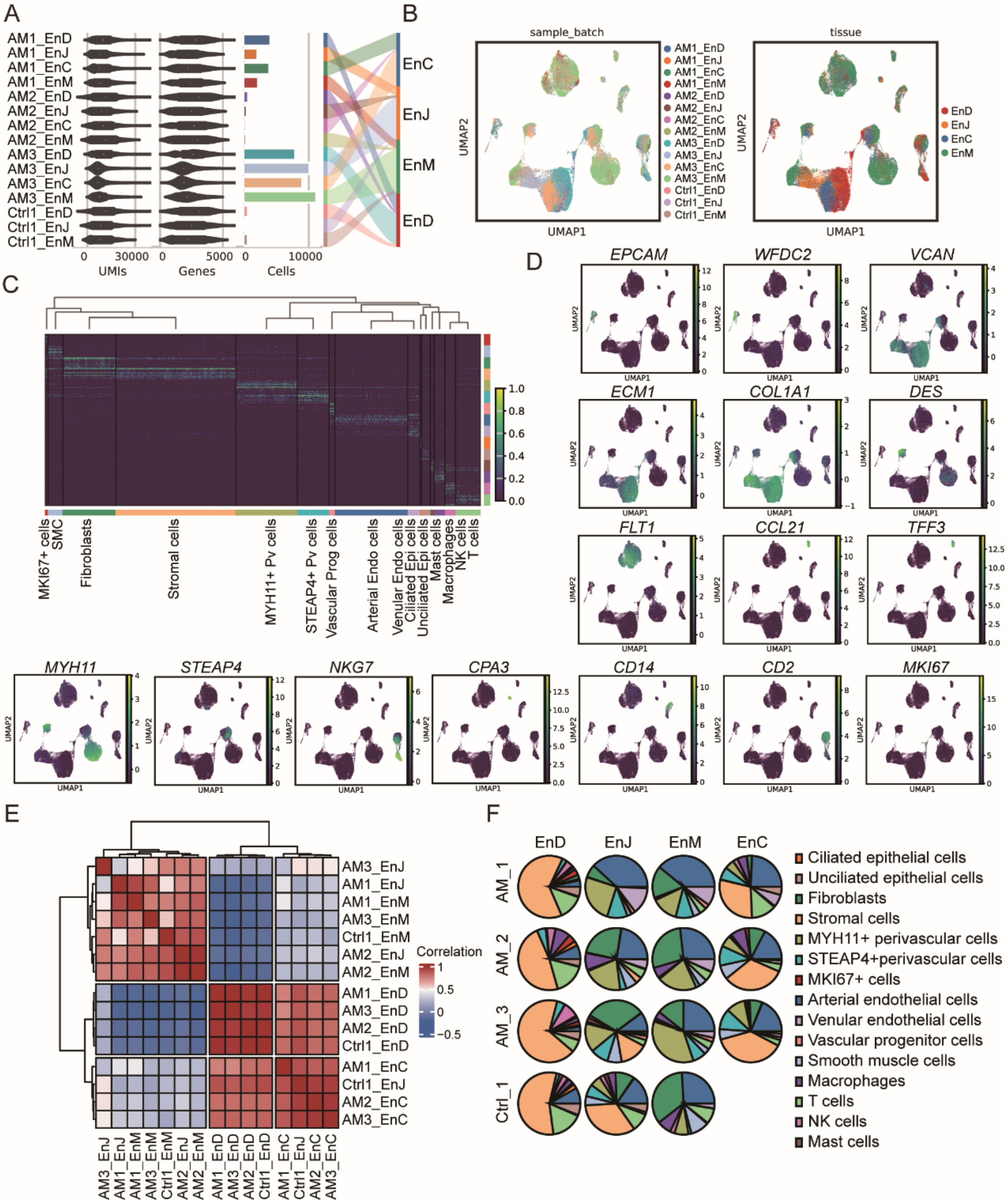

Figure S1. Quality control and standardization of scRNA-seq, expressions of marker genes and distribution of each sample in various uterine regions. (A) Diagram showing scRNA-seq metrics per sample (left) after quality control. These metrics indicate unique molecular identifier (UMIs) and total genes per cells across samples. The cord diagram (right) represents each sample in each tissue types. (B) Origin of droplet cells by sample (left) and tissue (right). (C) Heatmap revealing the scaled expression of differentially expressed genes for each cell type. (SMC: smooth muscle cells; Pv: perivascular; Prog: progenitor; Endo: endothelial; Epi: epithelial). (D) UMAP plot showing marker genes pattern in each cell type. (E) Correlation based on cluster frequencies, across all specimens profiled by scRNA-seq. (F) Pie chart represents major cell type proportions for each specimen.

Figure S2

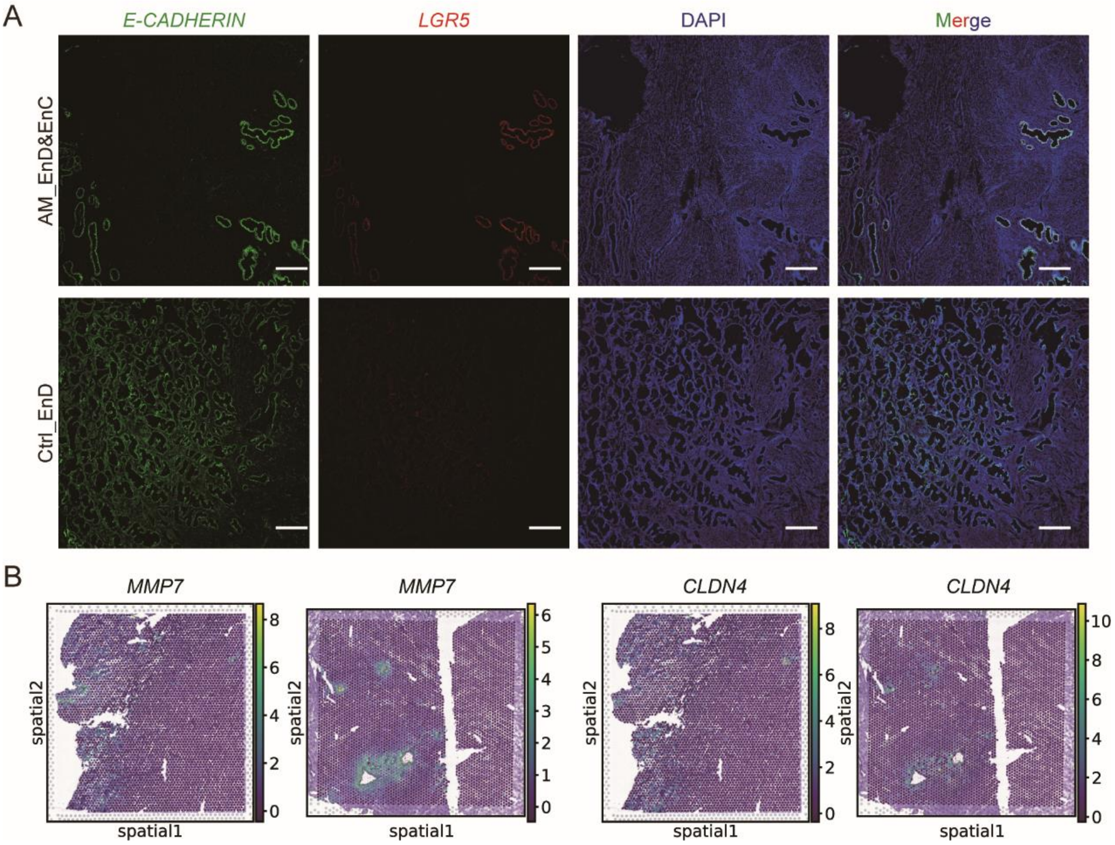

Figure S2. **Expression of characteristic genes of epithelial cells in adenomyosis lesions.** (A) Representative images of FISH for *LGR5* $^{+}$ cells in EnD and EnC of adenomyosis (upper panel), EnD of control (lower panel). Bar: 400 μm. (B) Visualization of *MMP7*$^{+}$ cells and *CLDN4*$^{+}$ cells in various regions of adenomyosis specimen by spatial transcriptomics.

Figure S3

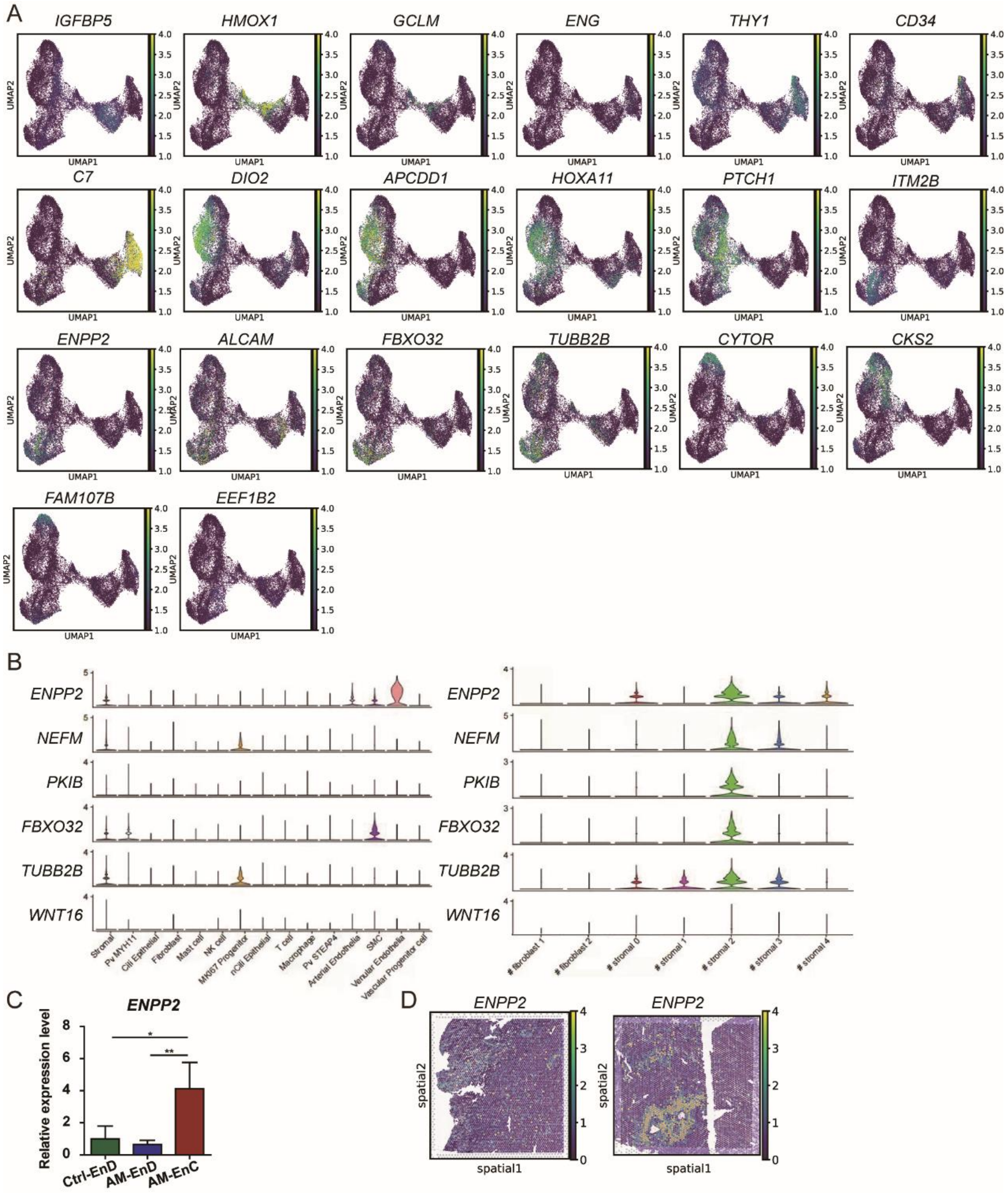

Figure S3. **Characteristic gene expression pattern in stromal subpopulations.** (A)Marker genes pattern in each stromal subclusters by UMAP plot. (B)The expression of marker genes of stromal 2 in all cell types and stromal subpopulations by Violin plot.

(C) mRNA expression levels of *ENPP2* for stromal 2 markers in Ctrl_EnD, AM_EnD and AM_EnC examined by qRT-PCR (n = 4 per group). Data are presented as the mean ± SEM, *$p < 0.05$, ** $p < 0.01$. (D) Visualization of *ENPP2*$^{+}$ cells in various regions of adenomyosis specimen by spatial transcriptomics.

FigureS4

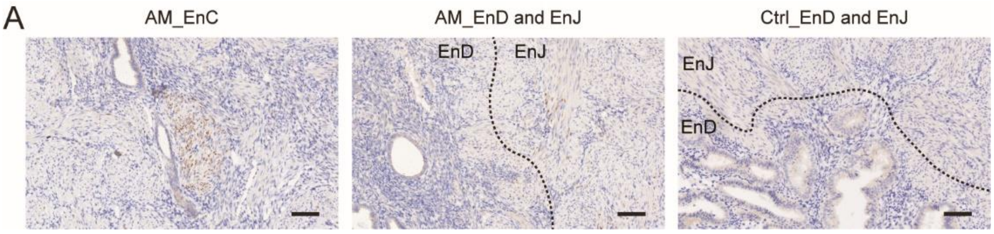

FigureS4.**The expression of WFDC1 in adenomyosis specimen by immunohistochemistry (IHC) staining.** Scale bar: 400 μm.

Figure S5

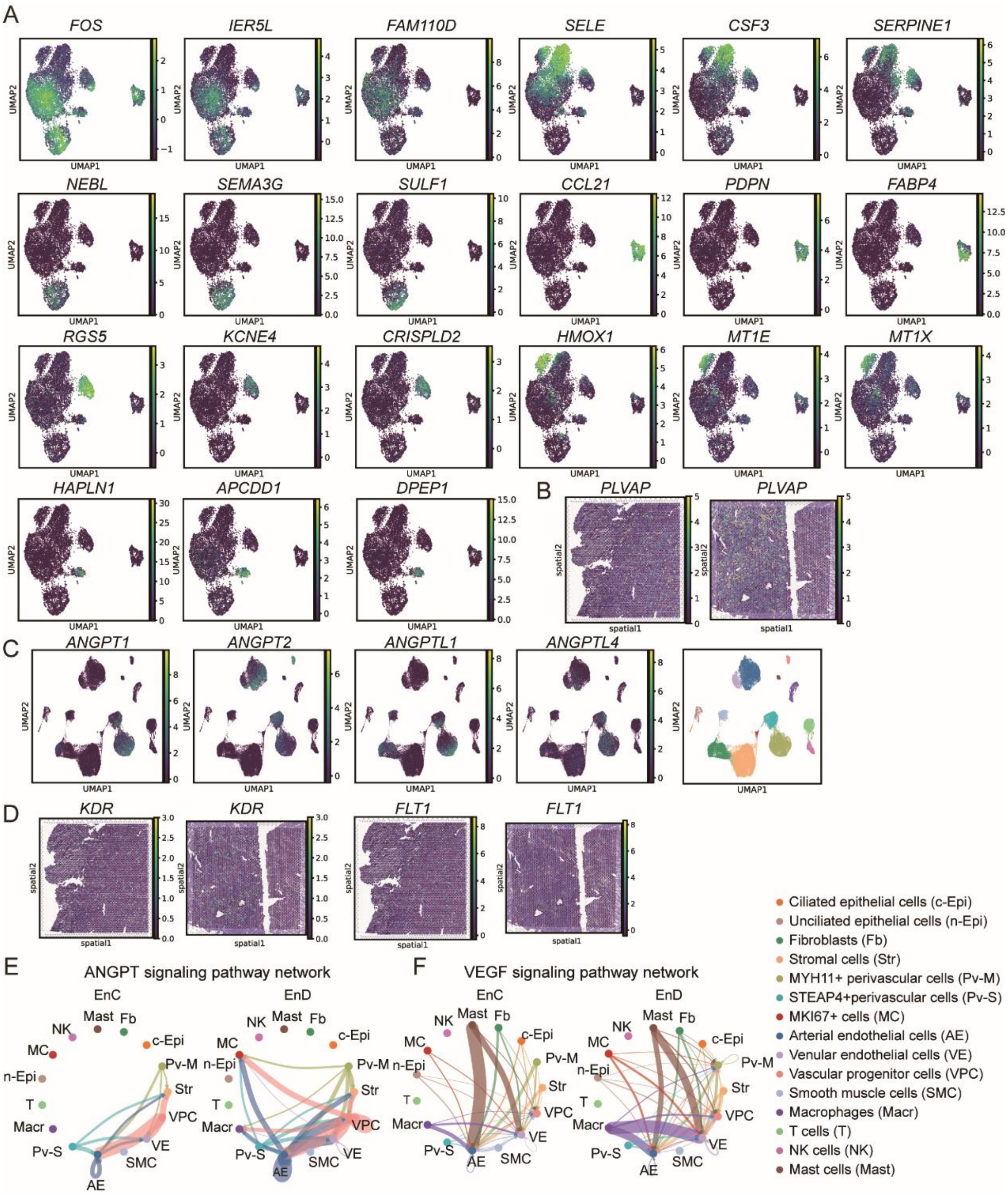

Figure S5. **Characteristic gene expression patterns of endothelial subpopulations in adenomyosis.** (A) Marker genes expression pattern in each endothelial subclusters

by UMAP plot. (B) Visualization of *PLVAP*$^{+}$ cells in spatial transcriptomics. (C) The expression of *ANGPT1*, *ANGPT2*, *ANGPTL1* and *ANGPTL4* in various cell types by UMAP plot. (D) Visualization of *KDR*$^{+}$ cells and *FLT1*$^{+}$ cells in spatial transcriptomics.

(E) Ligand-receptor pairs network of ANGPT signaling pathways in EnC and EnD. (F) Ligand-receptor pairs network of VEGF signaling pathways in EnC and EnD.

Figure S6

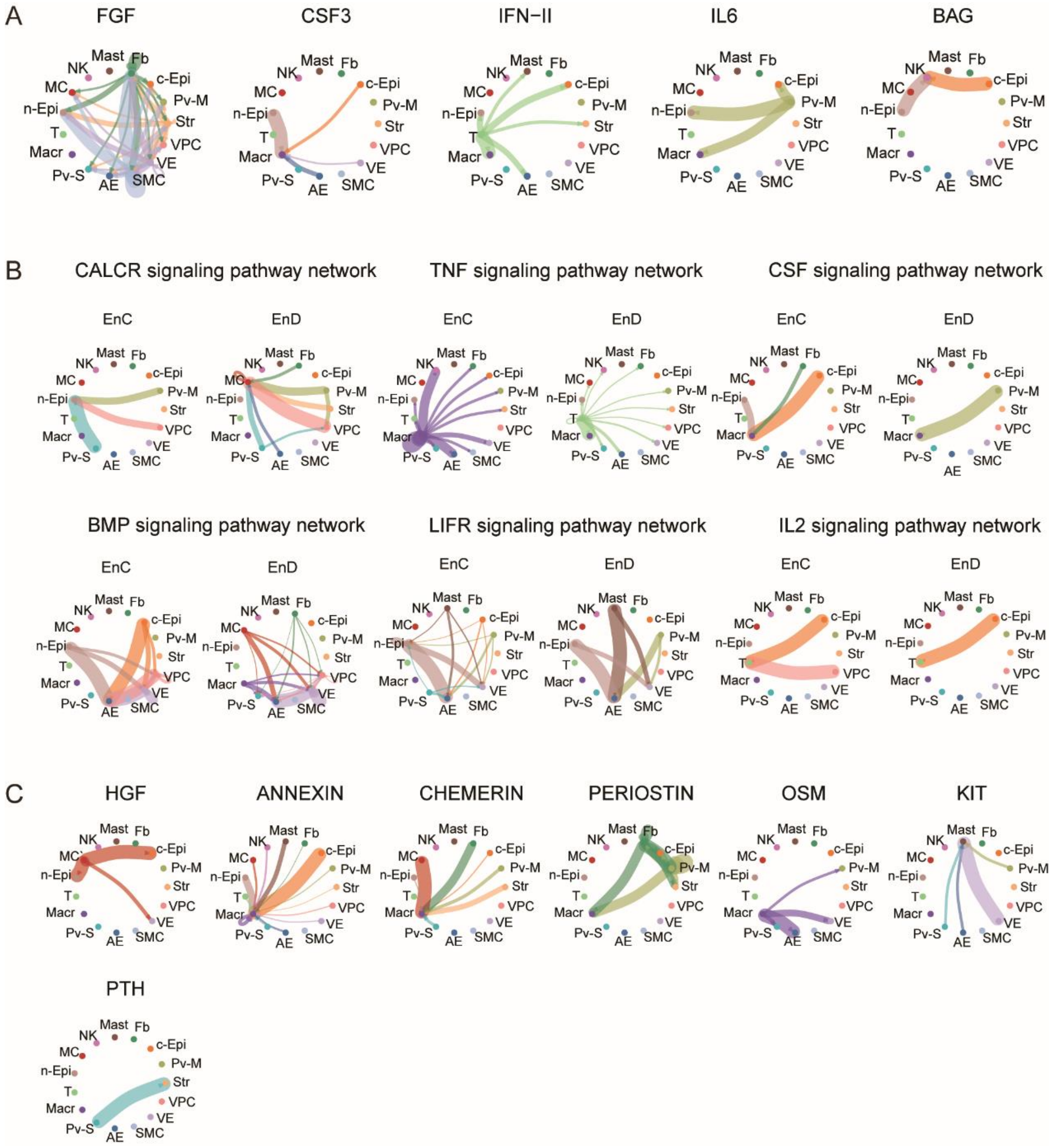

Figure S6. **Correlated signaling networks among different cell types in EnC and EnD of adenomyosis by CellPhoneDB.** (A) Network of intercellular interactions of specific signaling pathways for FGF, CSF3, IFN-II, IL6 and BAG in EnC. (B) Network of intercellular interactions of specific signaling pathways for CALCR, TNF, CSF, BMP, LIFR and IL2 in EnD and EnC. (C) Network of intercellular interactions of specific signaling pathways for HGF, ANNEXIN, CHEMERIN, PERIOSTIN, OSM, KIT and PTH in EnD.

Table S1. Clinical characteristics of adenomyosis patient samples used in this study. Related to Figure 1.

Disease	Location	Sample No.	Age	Pregnancy
Ademomyosis	Eutopic endometrium (EnD)	Sample A1-0	49	1-0-1-1
	Junctional zone (EnJ)	Sample A1-1		
	Adnomyophathy lesion (EnC)	Sample A1-2		
	Myometrium (EnM)	Sample A1-3		
Ademomyosis	Eutopic endometrium (EnD)	Sample A2-0	49	1-0-2-1
	Junctional zone (EnJ)	Sample A2-1		
	Adnomyophathy lesion (EnC)	Sample A2-2		
	Myometrium (EnM)	Sample A2-3		
Ademomyosis	Eutopic endometrium (EnD)	Sample A3-0	48	1-0-2-1
	Junctional zone (EnJ)	Sample A3-1		
	Adnomyophathy lesion (EnC)	Sample A3-2		
	Myometrium (EnM)	Sample A3-3		
uterine fibroids	Eutopic endometrium (EnD)	Sample C1-0	44	1-0-1-1
	Junctional zone (EnJ)	Sample C1-1		
	Myometrium (EnM)	Sample C1-2		

Table S2. stromal subpopulation in samples. Related to Figure 3.

	Fibroblast 1	Fibroblast 2	Stromal 0	Stromal 1	Stromal 2	Stromal 3	Stromal 4
EnD	36	0	5419	5	24	2723	347
EnJ	2919	48	468	606	74	114	751
EnC	406	249	139	45	2910	18	1490
EnM	104	2219	0	0	0	0	0

Table S3. Primer sequneces used for qRT-PCR in this study. Related to Figure 2, 3, 4.

Gene	Primer sequence
LGR5 rt F	CCTTGGCCCTGAACAAAATA
LGR5 rt R	ATTTCTTTCCCAGGGAGTGG
PKIB rt F	CCTCAAACTGGAGGCTCTCTCC
PKIB rt R	AGCACTCTTGATAGATTATGAGCC
ENPP2 rt F	TCAGAGGACGAATCAAAATGGG
ENPP2 rt R	CAGGTATGTCTTGAGTGTCAGG
WNT16 rt F	AAGTGAAGGCTGGCACTGG
WNT16 rt R	GGCAGTCTACTGACATCAACTTGG
APCDD1 rt F	GGAGTCACAGTGCCATCACATG
APCDD1 rt R	GGACCTTGTGATGAACTCTGGG
VWC2 rt F	GGAGTTCGTGGTGTCTCCATG
VWC2 rt R	CAAAGCAGTTTGGACCATTTTTGC
SFRP5 rt F	CACAAGTTCCCCCTGGACAA
SFRP5 rt R	TGTGCTCCATCTCACACTGG
GAPDH rt F	CTGCACCACCAACTGCTT
GAPDH rt R	TTCTGGGTGGCAGTGATG
